# Bis(μ-diphenyl­arsine-κ^2^ *As*:*As*)bis­[tetra­carbonyl­tungsten(0)]

**DOI:** 10.1107/S1600536810007592

**Published:** 2010-03-06

**Authors:** Edward R. T. Tiekink, James L. Wardell

**Affiliations:** a Department of Chemistry, University of Malaya, 50603 Kuala Lumpur, Malaysia; b Centro de Desenvolvimento Tecnológico em Saúde (CDTS), Fundação Oswaldo Cruz (FIOCRUZ), Casa Amarela, Campus de Manguinhos, Av. Brasil 4365, 21040-900 Rio de Janeiro, RJ, Brazil

## Abstract

The title compound, [W_2_(C_12_H_10_As)_2_(CO)_8_], features a diamond-shaped W_2_As_2_ core with a W—W distance of 3.0948 (7) Å. The coordination geometry for each W atom is based on a penta­gonal bipyramid within a As_2_C_4_W donor set, with carbonyl ligands defining the axial positions; the As atoms exist within distorted tetra­hedral C_2_W_2_ donor sets.

## Related literature

For information on the preparation, from *M*(CO)_6_ and *RE*_2_–*ER*_2_, and the IR spectra of [*M*_2_(*ER*_2_)_2_(CO)_8_] (*M* = Cr, Mo, or W; *E* = P or As; *R* = alkyl or aryl), see: Chatt & Thornton (1964 ▶). For other preparations and spectra of [W_2_(PPh_2_)_2_(CO)_8_], see: Shyu *et al.* (1987 ▶); Keiter & Madigan (1982 ▶); Keiter *et al.* (1989 ▶); Brown *et al.* (1995 ▶); Planinic & Matkovic-Calogovic (2001 ▶). For the crystal structure of [W_2_(PPh_2_)_2_(CO)_8_], see: Shyu *et al.* (1987 ▶).
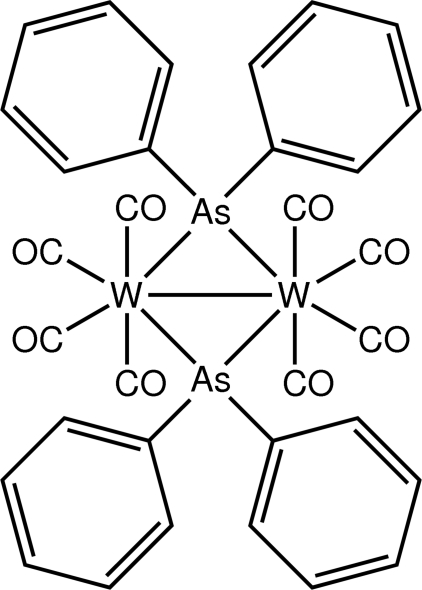


## Experimental

### 

#### Crystal data

[W_2_(C_12_H_10_As)_2_(CO)_8_]
*M* *_r_* = 1050.02
Monoclinic, 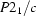

*a* = 9.7052 (4) Å
*b* = 20.1288 (7) Å
*c* = 16.6450 (7) Å
β = 102.835 (2)°
*V* = 3170.4 (2) Å^3^
*Z* = 4
Mo *K*α radiation
μ = 9.37 mm^−1^
*T* = 120 K
0.06 × 0.05 × 0.02 mm

#### Data collection

Nonius KappaCCD diffractometer
Absorption correction: multi-scan (*SADABS*; Sheldrick, 2007 ▶) *T*_min_ = 0.604, *T*_max_ = 0.746
25035 measured reflections
5544 independent reflections
4274 reflections with *I* > 2σ(*I*)
*R*_int_ = 0.105

#### Refinement

*R*[*F* ^2^ > 2σ(*F* ^2^)] = 0.061
*wR*(*F* ^2^) = 0.120
*S* = 1.62
5544 reflections
397 parameters
192 restraints
H-atom parameters constrained
Δρ_max_ = 2.56 e Å^−3^
Δρ_min_ = −1.28 e Å^−3^

### 

Data collection: *COLLECT* (Hooft, 1998 ▶); cell refinement: *DENZO* (Otwinowski & Minor, 1997 ▶) and *COLLECT*; data reduction: *DENZO* and *COLLECT*; program(s) used to solve structure: *SHELXS97* (Sheldrick, 2008 ▶); program(s) used to refine structure: *SHELXL97* (Sheldrick, 2008 ▶); molecular graphics: *ORTEP-3* (Farrugia, 1997 ▶) and *DIAMOND* (Brandenburg, 2006 ▶); software used to prepare material for publication: *publCIF* (Westrip, 2010 ▶).

## Supplementary Material

Crystal structure: contains datablocks global, I. DOI: 10.1107/S1600536810007592/hb5346sup1.cif

Structure factors: contains datablocks I. DOI: 10.1107/S1600536810007592/hb5346Isup2.hkl

Additional supplementary materials:  crystallographic information; 3D view; checkCIF report

## Figures and Tables

**Table 1:** Selected bond lengths (Å)

W1—C1	2.027 (13)
W1—C3	2.031 (14)
W1—C4	2.057 (15)
W1—C2	2.061 (14)
W1—As2	2.5597 (12)
W1—As1	2.5686 (13)
W1—W2	3.0948 (7)
W2—C6	1.983 (13)
W2—C8	2.027 (13)
W2—C7	2.033 (15)
W2—C5	2.048 (15)
W2—As2	2.5559 (13)
W2—As1	2.5652 (13)

**Fig. 1.**
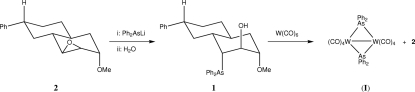
Reaction scheme leading to (I).

**Fig. 2.**
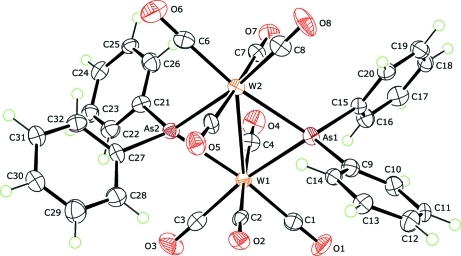
The molecular structure of (I) showing displacement ellipsoids at the 50% probability level.

## supplementary crystallographic information

### Comment

The title compound, (I), was isolated from a reaction mixture of W(CO)_6_ and the arsinosugar derivative, 4,6-benzylidene-3-deoxy-3-diphenylarsino-α-*D*-altropyranoside, 1 (Brown *et al.*, 1995), Fig. 1. The intention was to obtain a chiral tungsten complex, [W(CO)_4_(1)], but instead the species isolated indicated that the diphenylarsino fragment had been extracted from the chiral ligand with probable reformation of the epoxide reactant, 1, used in the preparation of 2 (Brown *et al.*, 1995), Fig. 1.

More direct routes to [M_2_(ER_2_)_2_(CO)_8_] compounds (M = Cr, Mo, or W; R = P or As) are available (Chatt & Thornton, 1964; Shyu *et al.*, 1987; Keiter & Madigan, 1982; Keiter *et al.*, 1989), including for [W_2_(AsMe_2_)_2_(CO)_8_] (Chatt & Thornton, 1964). Planinic & Matkovic-Calogovic (2001) reported the formation of [M_2_(PPh_2_)_2_(CO)_8_] (M = Mo or W) from a reaction mixture containing M(CO)_6_ and 1,4,8,11-tetrakis(methyldiphenylphosphino)-1,4,8,11-tetraazacyclotetradecane, another example of an abstraction of a Ph_2_M fragment from an elaborate and potential ligand.

The molecular structure of (I), Fig. 2, is constructed about planar four-membered W_2_As_2_ metallacycle [mean deviation = 0.012 (1) Å]. Even though the W–As distances span a narrow range [2.5559 (13) – 2.5686 (13) Å], the core has the shape of a diamond as the angles subtended at the W atoms [105.58 (4) and 105.79 (4) °] are greater than those subtended at the As atoms [74.15 (3) and 74.45 (3) °]. Each W atom exists within a pentagonal bipyramidal geometry defined by a W atom, two As atoms, and four carbonyl ligands. In this description, carbonyl ligands occupy axial positions: the C2–W1–C4 and C5–W2–C7 axial angles are 177.3 (5) and 177.7 (5) °, respectively. The As atoms exists in distorted tetrahedral C_2_W_2_ geometries [range of angles: 74.15 (3) to 124.4 (4) °]. The W1–W2 distance in (I) of 3.0948 (7) Å is longer than the corresponding distance [3.0256 (4) Å] in the isomorphous phosphino derivative (Shyu *et al.*, 1987).

### Experimental

A solution of tungsten hexacarbonyl (0.32 g, 1 mmol) and 4,6-benzylidene-3-deoxy-3-diphenylarsino-α-*D*-altropyranoside (0.49 g, 1 mmol) (Brown *et al.*, 1995) in THF (25 ml) was refluxed for 1 h and maintained at room temperature. Red plates of (I) formed slowly from this solution; m.pt. > 560 K. Found: C, 36.38; H, 2.27%;. C_32_H_20_As_2_O_8_W_2_ requires C, 36.60; H, 2.12%. IR (KBr) ν(CO) 2035(s), 1958(vs,br) cm^-1^.

### Refinement

The C-bound H atoms were geometrically placed (C–H = 0.95 Å) and refined as riding with *U_iso_*(H) = 1.2*U_eq_*(parent atom). The carbon atoms were refined with the ISOR command in SHELXL-97 (Sheldrick, 2008) to restrain the displacement parameters to be approximately isotropic. The maximum and minimum residual electron density peaks of 1.11 and 1.28 e Å^-3^, respectively, were located 0.99 Å and 0.84 Å from the W1 and H31 atoms, respectively.

### Crystal data

**Table:**

[W_2_(C_12_H_10_As)_2_(CO)_8_]	*F*(000) = 1960
*M**_r_* = 1050.02	*D*_x_ = 2.200 Mg m^−^^3^
Monoclinic, *P*2_1_/*c*	Mo *K*α radiation, λ = 0.71073 Å
Hall symbol: -P 2ybc	Cell parameters from 46502 reflections
*a* = 9.7052 (4) Å	θ = 2.9–27.5°
*b* = 20.1288 (7) Å	µ = 9.37 mm^−^^1^
*c* = 16.6450 (7) Å	*T* = 120 K
β = 102.835 (2)°	Plate, red
*V* = 3170.4 (2) Å^3^	0.06 × 0.05 × 0.02 mm
*Z* = 4	

### Data collection

**Table:**

Nonius KappaCCD diffractometer	5544 independent reflections
Radiation source: Enraf Nonius FR591 rotating anode	4274 reflections with *I* > 2σ(*I*)
10 cm confocal mirrors	*R*_int_ = 0.105
Detector resolution: 9.091 pixels mm^-1^	θ_max_ = 25.0°, θ_min_ = 2.9°
φ and ω scans	*h* = −11→10
Absorption correction: multi-scan (*SADABS*; Sheldrick, 2007)	*k* = −23→23
*T*_min_ = 0.604, *T*_max_ = 0.746	*l* = −19→19
25035 measured reflections	

### Refinement

**Table:**

Refinement on *F*^2^	Primary atom site location: structure-invariant direct methods
Least-squares matrix: full	Secondary atom site location: difference Fourier map
*R*[*F*^2^ > 2σ(*F*^2^)] = 0.061	Hydrogen site location: inferred from neighbouring sites
*wR*(*F*^2^) = 0.120	H-atom parameters constrained
*S* = 1.62	*w* = 1/[σ^2^(*F*_o_^2^) + (0.0272*P*)^2^] where *P* = (*F*_o_^2^ + 2*F*_c_^2^)/3
5544 reflections	(Δ/σ)_max_ = 0.001
397 parameters	Δρ_max_ = 2.56 e Å^−^^3^
192 restraints	Δρ_min_ = −1.28 e Å^−^^3^

### Special details

**Table:**

Geometry. All s.u.'s (except the s.u. in the dihedral angle between two l.s. planes) are estimated using the full covariance matrix. The cell s.u.'s are taken into account individually in the estimation of s.u.'s in distances, angles and torsion angles; correlations between s.u.'s in cell parameters are only used when they are defined by crystal symmetry. An approximate (isotropic) treatment of cell s.u.'s is used for estimating s.u.'s involving l.s. planes.
Refinement. Refinement of *F*^2^ against ALL reflections. The weighted *R*-factor wR and goodness of fit *S* are based on *F*^2^, conventional *R*-factors *R* are based on *F*, with *F* set to zero for negative *F*^2^. The threshold expression of *F*^2^ > 2σ(*F*^2^) is used only for calculating *R*-factors(gt) etc. and is not relevant to the choice of reflections for refinement. *R*-factors based on *F*^2^ are statistically about twice as large as those based on *F*, and *R*- factors based on ALL data will be even larger.

### Fractional atomic coordinates and isotropic or equivalent isotropic displacement parameters (Å^2^)

**Table:**

	*x*	*y*	*z*	*U*_iso_*/*U*_eq_	
W1	0.79610 (5)	0.22692 (2)	0.67301 (3)	0.02265 (16)	
W2	0.64555 (5)	0.17590 (2)	0.80661 (3)	0.02228 (16)	
As1	0.82208 (13)	0.11196 (6)	0.74172 (8)	0.0232 (3)	
As2	0.62376 (13)	0.29106 (6)	0.74031 (8)	0.0232 (3)	
O1	1.0294 (10)	0.1749 (5)	0.5819 (6)	0.044 (3)	
O2	0.5631 (9)	0.1541 (4)	0.5380 (5)	0.030 (2)	
O3	0.7593 (11)	0.3558 (5)	0.5629 (6)	0.050 (3)	
O4	1.0392 (10)	0.2917 (5)	0.8121 (6)	0.044 (3)	
O5	0.3671 (10)	0.1453 (4)	0.6717 (6)	0.034 (2)	
O6	0.4540 (11)	0.2428 (5)	0.9133 (6)	0.049 (3)	
O7	0.9180 (10)	0.2103 (4)	0.9456 (6)	0.039 (2)	
O8	0.6112 (10)	0.0395 (5)	0.8927 (7)	0.048 (3)	
C1	0.9463 (14)	0.1950 (6)	0.6147 (8)	0.031 (3)	
C2	0.6436 (13)	0.1799 (6)	0.5857 (8)	0.022 (3)	
C3	0.7752 (14)	0.3102 (7)	0.6025 (9)	0.033 (3)	
C4	0.9516 (15)	0.2696 (6)	0.7624 (8)	0.031 (3)	
C5	0.4670 (14)	0.1558 (6)	0.7184 (8)	0.026 (3)	
C6	0.5239 (13)	0.2183 (6)	0.8732 (8)	0.027 (3)	
C7	0.8194 (15)	0.1994 (6)	0.8951 (8)	0.028 (3)	
C8	0.6259 (14)	0.0879 (7)	0.8622 (8)	0.032 (3)	
C9	0.7768 (13)	0.0330 (6)	0.6756 (8)	0.025 (3)	
C10	0.8833 (14)	−0.0035 (6)	0.6561 (8)	0.027 (3)	
H10	0.9789	0.0090	0.6768	0.032*	
C11	0.8508 (13)	−0.0599 (5)	0.6048 (7)	0.023 (3)	
H11	0.9251	−0.0855	0.5919	0.028*	
C12	0.7164 (14)	−0.0775 (6)	0.5744 (8)	0.031 (3)	
H12	0.6961	−0.1162	0.5412	0.037*	
C13	0.6057 (13)	−0.0396 (6)	0.5912 (8)	0.027 (3)	
H13	0.5103	−0.0512	0.5679	0.032*	
C14	0.6369 (13)	0.0151 (5)	0.6421 (7)	0.023 (3)	
H14	0.5622	0.0409	0.6544	0.027*	
C15	0.9982 (12)	0.0849 (5)	0.8157 (7)	0.020 (3)	
C16	1.1266 (13)	0.1157 (6)	0.8160 (8)	0.031 (3)	
H16	1.1307	0.1513	0.7791	0.037*	
C17	1.2498 (15)	0.0944 (7)	0.8707 (9)	0.037 (3)	
H17	1.3373	0.1160	0.8719	0.044*	
C18	1.2434 (13)	0.0410 (6)	0.9237 (8)	0.028 (3)	
H18	1.3270	0.0255	0.9599	0.034*	
C19	1.1158 (14)	0.0110 (6)	0.9231 (8)	0.030 (3)	
H19	1.1110	−0.0247	0.9597	0.036*	
C20	0.9946 (14)	0.0328 (6)	0.8693 (8)	0.027 (3)	
H20	0.9071	0.0116	0.8692	0.032*	
C21	0.6862 (13)	0.3708 (6)	0.8044 (7)	0.024 (3)	
C22	0.6774 (14)	0.4307 (6)	0.7634 (8)	0.032 (3)	
H22	0.6413	0.4318	0.7055	0.038*	
C23	0.7196 (13)	0.4886 (6)	0.8045 (8)	0.031 (3)	
H23	0.7134	0.5295	0.7754	0.037*	
C24	0.7724 (14)	0.4870 (6)	0.8903 (8)	0.033 (3)	
H24	0.7993	0.5270	0.9199	0.039*	
C25	0.7847 (13)	0.4280 (6)	0.9309 (8)	0.025 (3)	
H25	0.8223	0.4262	0.9886	0.031*	
C26	0.7420 (13)	0.3707 (7)	0.8871 (8)	0.033 (3)	
H26	0.7519	0.3295	0.9157	0.040*	
C27	0.4386 (13)	0.3185 (6)	0.6796 (8)	0.026 (3)	
C28	0.3853 (14)	0.3008 (6)	0.5980 (8)	0.033 (3)	
H28	0.4433	0.2772	0.5687	0.040*	
C29	0.2473 (15)	0.3172 (6)	0.5588 (9)	0.040 (4)	
H29	0.2109	0.3044	0.5032	0.048*	
C30	0.1641 (14)	0.3521 (6)	0.6008 (8)	0.033 (3)	
H30	0.0706	0.3642	0.5738	0.039*	
C31	0.2163 (13)	0.3700 (6)	0.6833 (8)	0.028 (3)	
H31	0.1581	0.3938	0.7124	0.034*	
C32	0.3506 (13)	0.3532 (6)	0.7218 (8)	0.029 (3)	
H32	0.3854	0.3651	0.7778	0.034*	

### Atomic displacement parameters (Å^2^)

**Table:**

	*U*^11^	*U*^22^	*U*^33^	*U*^12^	*U*^13^	*U*^23^
W1	0.0259 (3)	0.0199 (3)	0.0226 (3)	−0.0006 (2)	0.0062 (2)	0.0000 (2)
W2	0.0227 (3)	0.0228 (3)	0.0208 (3)	−0.0012 (2)	0.0038 (2)	0.0014 (2)
As1	0.0220 (7)	0.0211 (6)	0.0251 (8)	−0.0009 (5)	0.0022 (6)	0.0006 (5)
As2	0.0273 (8)	0.0216 (6)	0.0210 (7)	−0.0009 (5)	0.0058 (6)	−0.0012 (5)
O1	0.041 (6)	0.052 (6)	0.045 (6)	0.010 (5)	0.024 (5)	−0.010 (5)
O2	0.033 (6)	0.030 (5)	0.022 (5)	−0.004 (4)	−0.005 (4)	−0.005 (4)
O3	0.070 (8)	0.032 (6)	0.045 (7)	−0.004 (5)	0.011 (6)	0.017 (5)
O4	0.035 (6)	0.039 (6)	0.058 (7)	−0.012 (5)	0.006 (5)	−0.016 (5)
O5	0.034 (6)	0.025 (5)	0.038 (6)	0.000 (4)	0.003 (5)	0.000 (4)
O6	0.058 (7)	0.047 (6)	0.047 (7)	−0.006 (5)	0.026 (6)	−0.007 (5)
O7	0.043 (6)	0.040 (5)	0.031 (6)	0.002 (5)	0.003 (5)	−0.004 (4)
O8	0.044 (6)	0.033 (5)	0.066 (8)	0.001 (5)	0.010 (6)	0.030 (5)
C1	0.030 (5)	0.034 (5)	0.026 (5)	0.002 (4)	0.002 (4)	−0.002 (4)
C2	0.023 (5)	0.022 (4)	0.023 (5)	0.001 (4)	0.011 (4)	−0.001 (4)
C3	0.035 (5)	0.033 (5)	0.033 (5)	0.000 (4)	0.012 (4)	−0.006 (4)
C4	0.033 (5)	0.029 (5)	0.030 (5)	−0.008 (4)	0.006 (4)	−0.001 (4)
C5	0.025 (5)	0.022 (4)	0.029 (5)	0.006 (4)	0.000 (4)	0.002 (4)
C6	0.025 (5)	0.031 (5)	0.026 (5)	−0.008 (4)	0.006 (4)	0.003 (4)
C7	0.027 (5)	0.030 (5)	0.026 (5)	0.003 (4)	0.002 (4)	0.003 (4)
C8	0.031 (5)	0.032 (5)	0.032 (5)	−0.004 (4)	0.004 (4)	0.002 (4)
C9	0.030 (5)	0.022 (4)	0.025 (5)	−0.001 (4)	0.005 (4)	0.000 (4)
C10	0.025 (5)	0.024 (4)	0.029 (5)	−0.001 (4)	0.001 (4)	−0.001 (4)
C11	0.024 (5)	0.020 (4)	0.026 (5)	0.008 (4)	0.005 (4)	0.001 (4)
C12	0.033 (5)	0.027 (5)	0.035 (5)	0.000 (4)	0.010 (4)	−0.004 (4)
C13	0.022 (5)	0.029 (5)	0.030 (5)	−0.003 (4)	0.007 (4)	−0.003 (4)
C14	0.028 (5)	0.019 (4)	0.020 (4)	0.001 (4)	0.004 (4)	−0.001 (4)
C15	0.017 (4)	0.020 (4)	0.021 (4)	−0.002 (4)	0.001 (4)	−0.002 (4)
C16	0.030 (5)	0.028 (5)	0.035 (5)	0.000 (4)	0.006 (4)	0.003 (4)
C17	0.035 (5)	0.039 (5)	0.036 (5)	−0.006 (4)	0.007 (4)	−0.004 (4)
C18	0.024 (5)	0.031 (5)	0.026 (5)	0.008 (4)	−0.003 (4)	−0.002 (4)
C19	0.034 (5)	0.030 (5)	0.027 (5)	0.001 (4)	0.005 (4)	0.003 (4)
C20	0.026 (5)	0.028 (5)	0.027 (5)	−0.001 (4)	0.009 (4)	0.001 (4)
C21	0.024 (5)	0.023 (4)	0.026 (5)	−0.008 (4)	0.007 (4)	−0.003 (4)
C22	0.032 (5)	0.035 (5)	0.030 (5)	−0.001 (4)	0.007 (4)	−0.003 (4)
C23	0.029 (5)	0.030 (5)	0.034 (5)	0.001 (4)	0.010 (4)	0.002 (4)
C24	0.033 (5)	0.031 (5)	0.037 (5)	0.002 (4)	0.013 (4)	−0.006 (4)
C25	0.027 (5)	0.029 (4)	0.020 (5)	0.000 (4)	0.005 (4)	−0.005 (4)
C26	0.029 (5)	0.034 (5)	0.035 (5)	0.001 (4)	0.005 (4)	0.003 (4)
C27	0.023 (5)	0.022 (4)	0.030 (5)	0.003 (4)	0.002 (4)	0.006 (4)
C28	0.033 (5)	0.036 (5)	0.032 (5)	0.001 (4)	0.010 (4)	0.002 (4)
C29	0.041 (5)	0.035 (5)	0.043 (5)	0.003 (4)	0.005 (4)	−0.001 (4)
C30	0.031 (5)	0.032 (5)	0.033 (5)	0.004 (4)	0.003 (4)	0.005 (4)
C31	0.024 (5)	0.026 (4)	0.032 (5)	0.002 (4)	0.002 (4)	0.002 (4)
C32	0.031 (5)	0.031 (5)	0.024 (5)	−0.003 (4)	0.005 (4)	−0.004 (4)

### Geometric parameters (Å, °)

**Table:**

W1—C1	2.027 (13)	C14—H14	0.9500
W1—C3	2.031 (14)	C15—C20	1.382 (16)
W1—C4	2.057 (15)	C15—C16	1.392 (16)
W1—C2	2.061 (14)	C16—C17	1.400 (18)
W1—As2	2.5597 (12)	C16—H16	0.9500
W1—As1	2.5686 (13)	C17—C18	1.399 (18)
W1—W2	3.0948 (7)	C17—H17	0.9500
W2—C6	1.983 (13)	C18—C19	1.376 (17)
W2—C8	2.027 (13)	C18—H18	0.9500
W2—C7	2.033 (15)	C19—C20	1.382 (18)
W2—C5	2.048 (15)	C19—H19	0.9500
W2—As2	2.5559 (13)	C20—H20	0.9500
W2—As1	2.5652 (13)	C21—C26	1.362 (18)
As1—C9	1.928 (12)	C21—C22	1.380 (17)
As1—C15	1.949 (12)	C22—C23	1.367 (17)
As2—C27	1.936 (13)	C22—H22	0.9500
As2—C21	1.948 (12)	C23—C24	1.407 (18)
O1—C1	1.143 (14)	C23—H23	0.9500
O2—C2	1.110 (14)	C24—C25	1.358 (17)
O3—C3	1.120 (15)	C24—H24	0.9500
O4—C4	1.136 (15)	C25—C26	1.377 (17)
O5—C5	1.120 (14)	C25—H25	0.9500
O6—C6	1.162 (14)	C26—H26	0.9500
O7—C7	1.146 (15)	C27—C32	1.405 (16)
O8—C8	1.122 (15)	C27—C28	1.389 (18)
C9—C10	1.364 (16)	C28—C29	1.393 (19)
C9—C14	1.396 (17)	C28—H28	0.9500
C10—C11	1.413 (16)	C29—C30	1.373 (18)
C10—H10	0.9500	C29—H29	0.9500
C11—C12	1.339 (17)	C30—C31	1.401 (18)
C11—H11	0.9500	C30—H30	0.9500
C12—C13	1.395 (16)	C31—C32	1.361 (17)
C12—H12	0.9500	C31—H31	0.9500
C13—C14	1.381 (16)	C32—H32	0.9500
C13—H13	0.9500		
			
C1—W1—C3	88.6 (5)	C11—C10—H10	120.1
C1—W1—C4	89.3 (5)	C12—C11—C10	120.6 (11)
C3—W1—C4	92.1 (5)	C12—C11—H11	119.7
C1—W1—C2	89.8 (5)	C10—C11—H11	119.7
C3—W1—C2	90.5 (5)	C11—C12—C13	120.5 (12)
C4—W1—C2	177.3 (5)	C11—C12—H12	119.7
C1—W1—As2	168.2 (4)	C13—C12—H12	119.7
C3—W1—As2	81.1 (3)	C14—C13—C12	119.0 (12)
C4—W1—As2	85.3 (4)	C14—C13—H13	120.5
C2—W1—As2	96.0 (3)	C12—C13—H13	120.5
C1—W1—As1	85.3 (4)	C13—C14—C9	120.8 (11)
C3—W1—As1	171.3 (4)	C13—C14—H14	119.6
C4—W1—As1	94.0 (4)	C9—C14—H14	119.6
C2—W1—As1	83.4 (3)	C20—C15—C16	119.1 (11)
As2—W1—As1	105.58 (4)	C20—C15—As1	118.4 (9)
C1—W1—W2	138.1 (4)	C16—C15—As1	122.5 (9)
C3—W1—W2	133.4 (3)	C15—C16—C17	120.0 (12)
C4—W1—W2	90.4 (3)	C15—C16—H16	120.0
C2—W1—W2	88.6 (3)	C17—C16—H16	120.0
As2—W1—W2	52.72 (3)	C16—C17—C18	119.6 (12)
As1—W1—W2	52.88 (3)	C16—C17—H17	120.2
C6—W2—C8	89.6 (5)	C18—C17—H17	120.2
C6—W2—C7	89.8 (5)	C19—C18—C17	119.9 (12)
C8—W2—C7	91.1 (5)	C19—C18—H18	120.0
C6—W2—C5	88.4 (5)	C17—C18—H18	120.0
C8—W2—C5	90.2 (5)	C18—C19—C20	120.0 (12)
C7—W2—C5	177.7 (5)	C18—C19—H19	120.0
C6—W2—As2	81.3 (3)	C20—C19—H19	120.0
C8—W2—As2	169.3 (4)	C15—C20—C19	121.3 (12)
C7—W2—As2	94.4 (3)	C15—C20—H20	119.3
C5—W2—As2	83.9 (3)	C19—C20—H20	119.3
C6—W2—As1	171.0 (4)	C26—C21—C22	117.9 (12)
C8—W2—As1	83.8 (4)	C26—C21—As2	123.9 (9)
C7—W2—As1	84.1 (3)	C22—C21—As2	118.2 (9)
C5—W2—As1	97.9 (3)	C23—C22—C21	121.2 (13)
As2—W2—As1	105.79 (4)	C23—C22—H22	119.4
C6—W2—W1	133.9 (3)	C21—C22—H22	119.4
C8—W2—W1	136.5 (4)	C24—C23—C22	119.3 (12)
C7—W2—W1	89.7 (3)	C24—C23—H23	120.3
C5—W2—W1	90.6 (4)	C22—C23—H23	120.3
As2—W2—W1	52.83 (3)	C23—C24—C25	119.8 (12)
As1—W2—W1	52.98 (3)	C23—C24—H24	120.1
C9—As1—C15	100.8 (5)	C25—C24—H24	120.1
C9—As1—W2	124.4 (4)	C26—C25—C24	119.1 (12)
C15—As1—W2	116.6 (3)	C26—C25—H25	120.5
C9—As1—W1	120.1 (4)	C24—C25—H25	120.5
C15—As1—W1	121.2 (3)	C25—C26—C21	122.6 (12)
W2—As1—W1	74.15 (3)	C25—C26—H26	118.7
C27—As2—C21	101.0 (5)	C21—C26—H26	118.7
C27—As2—W2	117.5 (3)	C32—C27—C28	118.7 (12)
C21—As2—W2	121.7 (4)	C32—C27—As2	118.5 (10)
C27—As2—W1	122.4 (4)	C28—C27—As2	122.6 (9)
C21—As2—W1	120.1 (3)	C29—C28—C27	120.5 (12)
W2—As2—W1	74.45 (3)	C29—C28—H28	119.7
O1—C1—W1	177.7 (11)	C27—C28—H28	119.7
O2—C2—W1	178.9 (10)	C30—C29—C28	119.7 (14)
O3—C3—W1	177.8 (13)	C30—C29—H29	120.1
O4—C4—W1	178.3 (12)	C28—C29—H29	120.1
O5—C5—W2	178.0 (11)	C29—C30—C31	120.4 (13)
O6—C6—W2	178.9 (12)	C29—C30—H30	119.8
O7—C7—W2	177.6 (11)	C31—C30—H30	119.8
O8—C8—W2	178.1 (12)	C32—C31—C30	119.7 (12)
C10—C9—C14	119.1 (11)	C32—C31—H31	120.1
C10—C9—As1	119.4 (10)	C30—C31—H31	120.1
C14—C9—As1	121.3 (9)	C31—C32—C27	120.9 (12)
C9—C10—C11	119.8 (12)	C31—C32—H32	119.5
C9—C10—H10	120.1	C27—C32—H32	119.5
			
C1—W1—W2—C6	166.9 (8)	W1—W2—As2—C21	−116.1 (4)
C3—W1—W2—C6	−15.7 (8)	C6—W2—As2—W1	175.4 (4)
C4—W1—W2—C6	77.5 (6)	C8—W2—As2—W1	−153 (2)
C2—W1—W2—C6	−105.0 (6)	C7—W2—As2—W1	86.3 (3)
As2—W1—W2—C6	−6.4 (5)	C5—W2—As2—W1	−95.3 (3)
As1—W1—W2—C6	172.3 (5)	As1—W2—As2—W1	1.14 (5)
C1—W1—W2—C8	−13.9 (8)	C1—W1—As2—C27	89.4 (19)
C3—W1—W2—C8	163.5 (8)	C3—W1—As2—C27	60.1 (6)
C4—W1—W2—C8	−103.3 (7)	C4—W1—As2—C27	152.9 (5)
C2—W1—W2—C8	74.2 (6)	C2—W1—As2—C27	−29.4 (5)
As2—W1—W2—C8	172.8 (6)	As1—W1—As2—C27	−114.2 (4)
As1—W1—W2—C8	−8.5 (6)	W2—W1—As2—C27	−113.0 (4)
C1—W1—W2—C7	77.5 (7)	C1—W1—As2—C21	−39.6 (19)
C3—W1—W2—C7	−105.1 (6)	C3—W1—As2—C21	−68.9 (6)
C4—W1—W2—C7	−11.9 (5)	C4—W1—As2—C21	23.9 (6)
C2—W1—W2—C7	165.6 (5)	C2—W1—As2—C21	−158.4 (5)
As2—W1—W2—C7	−95.8 (3)	As1—W1—As2—C21	116.8 (4)
As1—W1—W2—C7	82.8 (3)	W2—W1—As2—C21	118.0 (4)
C1—W1—W2—C5	−104.8 (6)	C1—W1—As2—W2	−157.6 (19)
C3—W1—W2—C5	72.6 (6)	C3—W1—As2—W2	173.1 (4)
C4—W1—W2—C5	165.8 (5)	C4—W1—As2—W2	−94.0 (3)
C2—W1—W2—C5	−16.7 (4)	C2—W1—As2—W2	83.6 (3)
As2—W1—W2—C5	81.9 (3)	As1—W1—As2—W2	−1.13 (5)
As1—W1—W2—C5	−99.4 (3)	C15—As1—C9—C10	−35.6 (11)
C1—W1—W2—As2	173.3 (6)	W2—As1—C9—C10	−168.7 (8)
C3—W1—W2—As2	−9.3 (5)	W1—As1—C9—C10	100.5 (10)
C4—W1—W2—As2	83.8 (4)	C15—As1—C9—C14	149.3 (10)
C2—W1—W2—As2	−98.6 (3)	W2—As1—C9—C14	16.2 (12)
As1—W1—W2—As2	178.63 (6)	W1—As1—C9—C14	−74.6 (10)
C1—W1—W2—As1	−5.4 (6)	C14—C9—C10—C11	−2.2 (18)
C3—W1—W2—As1	172.0 (5)	As1—C9—C10—C11	−177.4 (9)
C4—W1—W2—As1	−94.8 (4)	C9—C10—C11—C12	0.7 (19)
C2—W1—W2—As1	82.8 (3)	C10—C11—C12—C13	1.6 (19)
As2—W1—W2—As1	−178.63 (6)	C11—C12—C13—C14	−2.4 (18)
C6—W2—As1—C9	102 (2)	C12—C13—C14—C9	0.9 (18)
C8—W2—As1—C9	58.2 (6)	C10—C9—C14—C13	1.4 (18)
C7—W2—As1—C9	150.0 (6)	As1—C9—C14—C13	176.5 (9)
C5—W2—As1—C9	−31.2 (6)	C9—As1—C15—C20	−63.9 (10)
As2—W2—As1—C9	−117.0 (4)	W2—As1—C15—C20	73.8 (10)
W1—W2—As1—C9	−115.9 (4)	W1—As1—C15—C20	160.7 (8)
C6—W2—As1—C15	−24 (2)	C9—As1—C15—C16	115.7 (10)
C8—W2—As1—C15	−68.5 (5)	W2—As1—C15—C16	−106.6 (10)
C7—W2—As1—C15	23.3 (5)	W1—As1—C15—C16	−19.7 (11)
C5—W2—As1—C15	−157.8 (5)	C20—C15—C16—C17	−0.6 (18)
As2—W2—As1—C15	116.3 (4)	As1—C15—C16—C17	179.8 (9)
W1—W2—As1—C15	117.4 (4)	C15—C16—C17—C18	1.4 (19)
C6—W2—As1—W1	−142 (2)	C16—C17—C18—C19	−1.6 (19)
C8—W2—As1—W1	174.1 (4)	C17—C18—C19—C20	1.1 (18)
C7—W2—As1—W1	−94.1 (3)	C16—C15—C20—C19	0.1 (18)
C5—W2—As1—W1	84.7 (3)	As1—C15—C20—C19	179.7 (9)
As2—W2—As1—W1	−1.14 (5)	C18—C19—C20—C15	−0.4 (19)
C1—W1—As1—C9	−62.6 (6)	C27—As2—C21—C26	131.1 (11)
C3—W1—As1—C9	−17 (3)	W2—As2—C21—C26	−1.2 (12)
C4—W1—As1—C9	−151.6 (6)	W1—As2—C21—C26	−90.8 (11)
C2—W1—As1—C9	27.8 (5)	C27—As2—C21—C22	−50.9 (10)
As2—W1—As1—C9	122.1 (4)	W2—As2—C21—C22	176.8 (8)
W2—W1—As1—C9	121.0 (4)	W1—As2—C21—C22	87.2 (10)
C1—W1—As1—C15	64.5 (5)	C26—C21—C22—C23	−1.7 (18)
C3—W1—As1—C15	110 (3)	As2—C21—C22—C23	−179.9 (9)
C4—W1—As1—C15	−24.5 (5)	C21—C22—C23—C24	−0.3 (19)
C2—W1—As1—C15	154.9 (5)	C22—C23—C24—C25	2.0 (19)
As2—W1—As1—C15	−110.7 (4)	C23—C24—C25—C26	−1.5 (18)
W2—W1—As1—C15	−111.9 (4)	C24—C25—C26—C21	−0.7 (19)
C1—W1—As1—W2	176.4 (4)	C22—C21—C26—C25	2.3 (19)
C3—W1—As1—W2	−138 (3)	As2—C21—C26—C25	−179.7 (9)
C4—W1—As1—W2	87.4 (4)	C21—As2—C27—C32	−43.6 (10)
C2—W1—As1—W2	−93.2 (3)	W2—As2—C27—C32	91.2 (9)
As2—W1—As1—W2	1.13 (5)	W1—As2—C27—C32	179.6 (8)
C6—W2—As2—C27	−65.8 (6)	C21—As2—C27—C28	141.5 (10)
C8—W2—As2—C27	−34 (2)	W2—As2—C27—C28	−83.7 (10)
C7—W2—As2—C27	−154.8 (5)	W1—As2—C27—C28	4.8 (12)
C5—W2—As2—C27	23.5 (5)	C32—C27—C28—C29	0.1 (18)
As1—W2—As2—C27	120.0 (4)	As2—C27—C28—C29	174.9 (10)
W1—W2—As2—C27	118.9 (4)	C27—C28—C29—C30	1.0 (19)
C6—W2—As2—C21	59.3 (6)	C28—C29—C30—C31	−1.3 (19)
C8—W2—As2—C21	91 (2)	C29—C30—C31—C32	0.6 (19)
C7—W2—As2—C21	−29.8 (5)	C30—C31—C32—C27	0.5 (18)
C5—W2—As2—C21	148.6 (5)	C28—C27—C32—C31	−0.8 (18)
As1—W2—As2—C21	−114.9 (4)	As2—C27—C32—C31	−175.9 (9)
